# Impact of a livelihood promotion program on income generation and food consumption among ultra-poor households in rural Bangladesh

**DOI:** 10.1080/16549716.2022.2031595

**Published:** 2022-04-25

**Authors:** Paige Volpenhein, Hee Sun Kim, Yunjeong Kim, MD. Iqbal Hussein, Jaganmay Biswas, Sunwoo Byun, Yunhee Kang

**Affiliations:** a Department of Food, Nutrition, and Health, Johns Hopkins Bloomberg School of Public Health, Baltimore, MD, USA; b Department of Food and Nutrition, College of Human Ecology, Seoul National University, South Korea; c World Vision Bangladesh, Dhaka, Bangladesh; d World Vision International, London, UK; e Center for Human Nutrition, Johns Hopkins Bloomberg School of Public Health, Baltimore

**Keywords:** Ultra-poor population, poverty eradication, graduation approach, livelihood program, food security

## Abstract

**Background:**

Bangladesh has achieved notable economic progress in recent decades while economic inequality increased. Special attention is warranted on the ultra-poor population of the country. An 18 month-long economic development program, designed based on an ultra-poor graduation approach, was implemented to alleviate poverty and improve child nutrition in rural Bangladesh.

**Objective:**

The study examined the impact of livelihood components of an economic development program on outcomes related to poultry/crop production, consumption, and income generation among the ultra-poor throughout quarterly follow-ups.

**Methods:**

This secondary data analysis used the monitoring records of 2960 poor or ultra-poor households receiving assets of (1) 9–26 ducks (n = 2125), (2) 11 chickens (n = 872), and/or (3) vegetable seeds (n = 2407). Data measuring the production of assets, income generation, and consumption of assets were collected quarterly throughout 2019. To examine a one-year-long trend in participation, production, income generation, and consumption of assets, a one-way analysis of variance was conducted across the follow-ups. Additional analyses of annual income and consumption comparing duck and chicken groups were performed using linear regression models.

**Results:**

The number of poultry assets per household decreased between the April– June and July–Sep follow-ups, while consumption of poultry and vegetable assets increased during the monsoon season (p < 0.001 for all). The vegetable production reflected seasonal fluctuations, where the lowest production and income were reported during the monsoon and pre-monsoon seasons. We observed increasing voluntary adoption of poultry farming among the non-asset group for both duck and chicken over the follow-ups (p < 0.001 for all). The households provided with duck assets gained a greater mean annual income compared to the households provided with chicken assets.

**Conclusions:**

Our findings highlight opportunities for strengthening the ultra-poor graduation approach on livelihood promotion in future scale-up in rural Bangladesh.

## Background

Bangladesh has achieved significant economic progress, with its national GDP jumping approximately 200% between 2009 and 2019, a result of the joint effort of international and national sectors [1]. Numerous poverty eradication strategies have been tried in Bangladesh, including micro-credit projects in the early 1980s [2] and economic development (ED) programs, such as lump sum cash transfer or livelihood development programs [3]. While overall national economic growth has driven a reduction in national poverty rates, from 48.9% in 2000 to 23.2% in 2016 [4], the Gini index of Bangladesh jumped concomitantly, from 25.9% to 32.4% between 1983 and 2016 [5]. The increasing discrepancy between the rate of poverty decline and economic inequality emerged as a continuing challenge, alarming special attention on the ultra-poor population in Bangladesh. Nationally, the disproportionate economic opportunities may hinder the country’s progress [6]. Thus, careful multi-dimensional efforts are needed to narrow the economic gap within the country.

**Figure 1.**
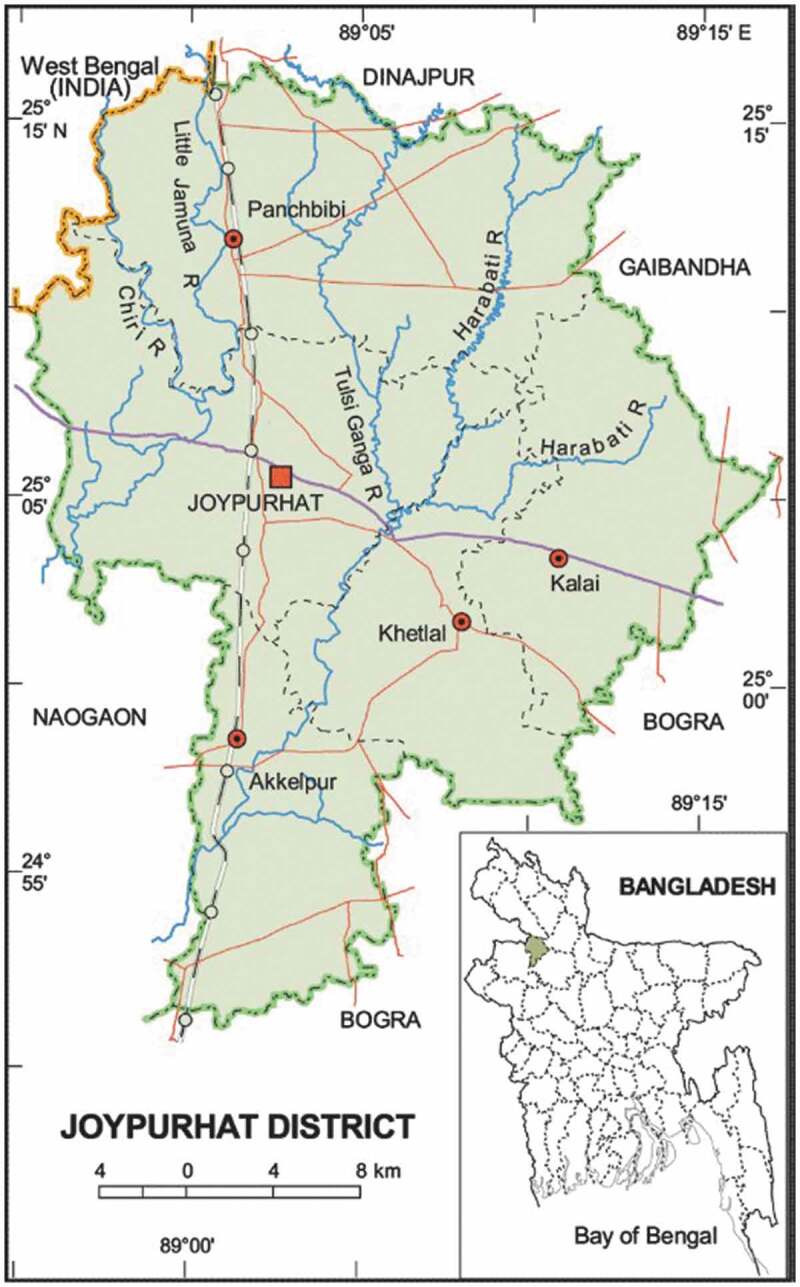
Map of study area.

**Figure 2.**
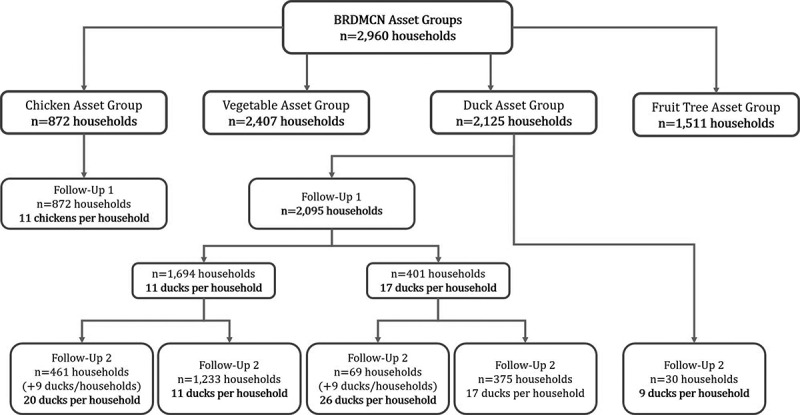
Household asset provisions.

**Table 1.** Asset management characteristics of the participating beneficiaries in ED program in Bangladesh over 12-months follow up

		3mo F/U (JAN-MAR 2019)	6mo F/U (APR-JUN 2019)	9mo F/U (JUL-SEP 2019)	12mo F/U (OCT-DEC 2019)	
Asset		Mean (±SD)	Mean (±SD)	Mean (±SD)	Mean (±SD)	p-value^c^
Duck ^a^	Total household^b^	1228	1232	1223	1233	
	Asset per household^b^	9.2 (5.7)	14.9 (10.0)	8.0 (8.1)	6.2 (5.8)	<0.001
	Newly born^b^	2.5 (4.8)	2.3 (5.1)	1.1 (3.5)	1.2 (3.6)	<0.001
	Newly purchased^b^	1.0 (2.8)	1.0 (3.0)	1.1 (6.3)	0.9 (2.1)	0.57
	Died^b^	3.7 (2.5)	3.1 (3.3)	4.5 (5.4)	1.8 (3.6)	<0.001
	Meat consumed^b^	0.2 (0.6)	0.6 (1.3)	1.9 (2.3)	1.2 (2.4)	<0.001
	Egg consumed^b^	73.0 (99.8)	59.1 (119.8)	47.2 (73.8)	40.6 (59.9)	<0.001
	Asset sold^b^	1.5 (3.3)	0.9 (2.3)	2.7 (4.7)	1.0 (3.0)	<0.001
	Income (TK) ^b^	1009 (1208)	694 (1207)	971 (1334)	582 (1055)	<0.001
Chicken	Total household^b^	856	872	861	872	
	Asset per household^b^	9.0 (5.9)	16.9 (13.6)	10.7 (10.8)	8.2 (8.7)	<0.001
	Newly born^b^	1.4 (4.3)	2.2 (5.2)	2.5 (5.1)	3.2 (5.6)	<0.001
	Newly purchased^b^	1.8 (3.5)	1.6 (7.3)	1.4 (4.8)	1.2 (3.1)	0.19
	Died^b^	4.0 (3.3)	4.0 (4.1)	4.3 (4.3)	2.5 (3.4)	<0.001
	Meat consumed^b^	0.5 (1.2)	0.9 (2.1)	2.8 (3.8)	2.4 (4.0)	<0.001
	Egg consumed^b^	13.2 (35.0)	25.4 (57.5)	35.9 (69.5)	23.7 (48.4)	<0.001
	Asset sold^b^	0.9 (2.8)	0.7 (2.1)	3.2 (5.7)	2.0 (4.1)	<0.001
	Income (TK) ^b^	292 (748)	333 (752)	753 (1244)	544 (1129)	<0.001
Vegetable	Total household ^b^	1709	2407	2358	2407	
	Produced (kg) ^b^	26.8 (41.2)	14.7 (39.8)	17.0 (39.3)	20.1 (47.7)	<0.001
	Consumed (kg) ^b^	15.0 (20.4)	7.8 (12.6)	10.7 (14.7)	12.0 (19.8)	<0.001
	Sold (kg) ^b^	14.2 (40.0)	6.9 (32.2)	6.3 (23.8)	8.1 (36.4)	<0.001
	Income (TK) ^b^	256 (730)	92 (391)	85 (337)	118 (466)	<0.001

Abbreviations: ED, Economic Development; SD, Standard Deviation; TK, Bangladeshi Taka

^a^Households with 11 ducks as the initial asset in the ED program.

^b^One-way analysis of variance (ANOVA) was conducted across the quarterly follow-ups.

^c^P-value was generated by chi-square test.

**Table 2.** Asset maintenance and new adoption of ducks, chicken, and vegetables of the participating beneficiaries in ED program in Bangladesh over 12-months follow up

		3mo F/U (JAN-MAR 2019)	6mo F/U (APR-JUN 2019)	9mo F/U (JUL-SEP 2019)	12mo F/U (OCT-DEC 2019)	p-value ^a^
Characteristics	n	n (%)	n (%)	n (%)	n (%)	n (%)
Households maintaining ducks among duck group	2095	2032 (97.0)	2052 (97.9)	1829 (87.3)	1770 (84.5)	<0.001
Households newly adopting ducks among non-duck group	865	14 (1.6)	61 (7.1)	97 (11.2)	156 (18.3)	<0.001
Households maintaining chicken	872	801 (91.9)	818 (93.8)	728 (83.5)	696 (79.8)	<0.001
Households newly adopting chicken from non-chicken group	2088	146 (7.0)	217 (10.4)	343 (16.4)	974 (46.6)	<0.001
Households producing vegetables	2403	N/A	1584 (65.9)	1671 (69.5)	1773 (73.8)	<0.001

Abbreviations: ED, Economic Development;

^a^P-value was generated by chi-square test.

**Table 3.** Annual income and consumption of the participating beneficiaries with 11 ducks and 11 chicken in ED program in Bangladesh

		n	Mean (±SD)	Median (IQR)	Min, Max	Crude log transformed β coefficient(95%CI)	p value	Adjusted log transformed β coefficient(95%CI) ^a^	p-value
Annual Income (TK)	Duck	1218	1480 (1892)	900 (0, 2100)	0, 16,000	1.18 (1.07–1.29)	0.001	1.15 (1.05–1.26)	0.003
	Chicken	845	1283 (1655)	650 (0, 1950)	0, 11,700	REF		REF	
	Duck Egg	1203	1778 (2253)	1045 (300, 2490)	0, 22,850	2.22 (2.22–2.51)	<0.001	2.21 (1.97–2.49)	<0.001
	Chicken Egg	823	657 (1376)	80 (0, 700)	0, 10,248	REF		REF	
	Duck + Egg	1200	3267 (3246)	2400 (1000, 4650)	0, 24,650	1.68 (1.52–1.86)	<0.001	1.65 (1.50–1.82)	<0.001
	Chicken + Egg	822	1930 (2321)	1175 (145, 2878)	0, 14,400	REF		REF	
Annual consumption	Duck	1222	3.8 (3.7)	3 (1, 5)	0, 32	0.08 (0.06–0.12)	<0.001	0.09 (0.06–0.13)	<0.001
	Chicken	846	6.6 (6.0)	5 (2, 9)	0, 39	REF		REF	
	Duck Egg	1196	224 (235)	170 (72, 295)	0, 2190	2.49 (2.27–2.73)	<0.001	2.40 (2.19–2.62)	<0.001
	Chicken Egg	820	101 (139)	49 (10, 133)	0, 740	REF		REF	

^a^Adjusted for village location

A total of 31% of children under five years are reported to suffer from stunting in Bangladesh [7] Furthermore, a multi-year analysis of Bangladeshi dietary patterns found that recent improvements in the agricultural system rather decreased the household dietary diversity, increasing adherence to monotonous diets [8]. The majority of the consumption is from cereals, where 58% is from rice alone. Thus, the subsequent insufficiency of protein, fat, vitamin, and mineral sources may exacerbate the malnutrition status in Bangladesh [9]. The imbalanced diet and poor dietary diversity may reflect the insufficient domestic production of animal sources and micronutrient rich foods. Of note, the poorest households were reported to be more cereal-dependent than the richest group [10].

Despite many efforts for poverty eradication in Bangladesh, marginalized populations were often missed by conventional development programs. The Graduation Approach (GA), developed to address ultra-poor populations in Bangladesh, specifically focuses on the limitations and needs of the ultra-poor households to optimize program impact. A holistic approach, including income security, livelihood management, health, and education, may yield synergistic outcomes to improve family resilience and empowerment [11]. Moreover, education on asset management, saving practices, and social integration are essential to achieve sustainable affirmative impacts for future generations.

While many studies have highlighted the effectiveness of the GA across a variety of Low- and Middle-income Countries (LMICs), some authors suggest that the actual benefits of these programs have been inflated in rhetoric [12]. Although the results of GA studies showed real and relevant improvements to the state of household economies, there was questionable support for whether these improvements were significant and sustainable enough to provide the ‘big push’ necessary for households to graduate from poverty [12]. More research is still needed to strengthen the impact bonds and further assure the long-term effectiveness for poverty eradication efforts.

The Bangladesh Rajshahi Division Maternal and Child Nutrition (BRDMCN) program by World Vision (WV) included activities in the realms of economic development (ED) and social behavior change communications to improve health and nutrition. The ED pillar of the BRDMCN program adopted the ultra-poor GA [13] to meet the immediate needs of the ultra-poor population, as well as to build capacity to promote long-term economic gains. The GA takes a holistic approach based on the four primary pillars: social protection, livelihoods promotion, financial inclusion, and social empowerment [3,11].

A recent meta-analysis compared the cost-effectiveness of three different forms of social protection interventions (livelihood development, lump-sum unconditional cash transfers, and graduation programs) [14]. The study found more pronounced long-term positive impacts on saving, household assets, and productive assets from the graduation initiatives. The meta-average of the ratios of impact on annual consumption to cost was also calculated, and the graduation programs had a greater benefit–cost ratio of 0.11, compared to 0.09 for livelihood programs [15]. A pilot study of randomized trials among 10,495 households in Ethiopia, Ghana, Honduras, India, Pakistan, and Peru found significant positive impacts three years after program completion on food security, dietary quality, income generation, and women empowerment [16]. Another multi-year analysis on the impact of the graduation program found 7-year-long sustained positive impacts on social protection indicators, including access to secure employment, poverty reduction, women empowerment, and child well-being [17].

The primary goal of WV’s graduation program is to place ultra-poor families on an upward economic trajectory, providing them with a means of self-employment that will allow them to be self-reliant. Evaluating the impact of ED programs conducted by WV in supported communities is vital to program success and sustainability. Yet, limited studies have examined the impact of ED programs implemented by WV in Bangladesh.

To track the success of the livelihood components of its program, this study, using the monitoring data of a GA program, examines the component related to poultry/crop production, consumption, and income generation among beneficiary households in rural Bangladesh. Specifically, the study compares outcomes across quarterly follow-ups and asset group types to determine the program impact and inform best practices for strengthening programs. Primary outcomes include asset management, income, and asset maintenance, measured over four quarterly follow-ups among beneficiary households from Jan to Dec 2019. Consumption of assets is a secondary outcome, which is considered within asset management for the seasonal analysis and is analyzed on its own for the asset group analysis.

## Methods

### Study settings

The present study is a secondary data analysis using the program monitoring records of three Upazilas in the Joypurhat District of the Rajshahi Division, located in rural Bangladesh (Figure 1). WV had a longstanding presence in the region and had made important strides in reducing malnutrition through rural development programs. Based on nutrition improvements seen in a few communities supported by WV in the Joypurhat District, the District Civil Surgeon requested to expand nutrition programs in communities without existing activities. The BRDMCN program was designed and implemented from March 2018 to December 2020 jointly by World Vision Bangladesh (WVB) and World Vision Korea (WVK) [18]. The goals of the BRDMCN program were to improve household livelihood and promote infant feeding practices and child nutrition in rural Bangladesh. The BRDMCN included two main strategic components: a social behavior change (SBC) component and an ED component. Detailed program information is described elsewhere [18].

### ED program

WV has developed a comprehensive training guide for the assessment, design, and evaluation of livelihood programs. The operation period of the graduation program is generally 18 to 24 months long [3], and the program provides training in technical skills, asset transfers, enterprise development, and saving & planning strategies for ultra-poor households [11]. The present study specifically focuses on the livelihood component of GA within ‘ED’ component. However, to align with the linked studies from the parental project [18], the term ‘ED’ will be used to describe the overall impact of the livelihood component in the present study.

The BRDMCN team, community facilitators, and community leaders screened and selected the ED program beneficiaries. First, 15,489 ultra-poor households were selected based on the wealth ranking index by the WVB. Data on ownership of household durable goods, dwelling characteristics, potable water source, and sanitation facilities were collected. The scores of each asset were summed to generate the total score of individual households, then ranked into quintiles. The participant’s capability, interest, and living environments for asset distribution were also assessed. Based on the acquired information, participants were assigned to either agricultural or non-agricultural groups. Out of 15,489 households, a total of 2960 ED households with young children or currently pregnant members were selected as beneficiaries, including 1762 households with children under two years old and 1198 households with pregnant women [18]. Of the 2960 households, 1332 were categorized as poor and 1628 households were considered ultra-poor by the project’s wealth ranking process.

After consultation with community facilitators, the enrolled 2960 households were matched to appropriate asset type(s). The consultation considered household resources, skillsets, and motivation. Groupings resulted in households receiving ducks (n = 2125), chicken (n = 870), vegetable seeds and garden training (n = 2075), and/or 1 to 4 nursery fruit trees (n = 1511). Various trainings – including livelihoods technical training, business training, financial literacy training, and social empowerment mentoring – were provided for the participants. Gardening trainings included two days of gardening instruction for households receiving vegetable seeds. Business operation trainings were held only for participants with prior microbusiness experience (n = 230). Quarterly education sessions on monetary savings techniques were included for all program participants (n = 2960). In addition to the quarterly trainings, community facilitators held monthly meetings on savings in each community. Additional program technical support was provided by the Department of Agriculture Extension and Livestock. The monitoring records of households receiving nursery trees (n = 1511) are excluded from analysis at one-year follow-up due to a longer period of nurturing prior to income generation.

### Data collection

A collaborative M&E system was established by a group of researchers and the project team to collect monitoring and outcome data of the BRDMCN [18]. The objectives of the monitoring system were to gather the data needed to evaluate the intended outcomes and identify households in need of additional support. The data was collected via paper forms by 285 community facilitators for 2960 households. The data from forms submitted to a WV M&E officer was transferred to an excel-based tracking system.

The monitoring data collected for households receiving ducks or chickens were 1) asset management: total number of assets, number newly born, number newly purchased, and number died; 2) consumption: consumed number of duck/chicken meat and eggs; and 3) income: income by selling duck/or chicken and eggs. For groups receiving vegetable assets, the following data were collected: 1) amount produced, amount consumed, and amount sold and 2) income: income from selling vegetables.

The community facilitators visited each household to measure and record relevant data on a quarterly basis throughout 2019 (3-month follow-up: Jan–March; 6-month follow-up: April–June; 9-month follow-up: July–Sep; 12-month follow-up: Oct–Dec). During each visit, monitoring staff assessed the need for additional training, resources, or assets.

### Variable construction

Primary Outcomes:

1. Asset management: includes number of assets present, born, purchased, died, and consumed.
2. Income generation: generated revenue from selling assets (poultry meat, eggs, and vegetables). 85 TK is approximately equivalent to $1 USD.
3. Asset maintenance: indicated the percentage of households with assets present (>0 assets) at each follow-up. The asset maintenance was also assessed among counter asset group who may have invested in further assets different than their initial group (defined as households that did not initially receive the asset).

Secondary Outcomes:

Asset consumption: assets consumed by household.

For primary and secondary outcomes, the number of poultry and eggs and the amount of vegetable (kg) were recorded.

### Statistical analysis

A longitudinal analysis was conducted to test the association between categories (asset groups) and time (follow-up periods). All statistical analyses were conducted via Stata (Stata/IC 16.1, StataCorp); all tests were two-sided, and the significance level P < 0.05 was considered significant.

For trend analysis of asset management, the dataset was reshaped to a longitudinal format, and one-way ANOVAs were conducted to compare the means and standard deviations (SDs) across the quarterly follow-ups of asset characteristics, including participating households, assets per household, newly born, newly purchased, asset deaths, consumption, number sold, and income. To correct for the distribution of additional duck assets at 6-month follow-up, and to keep outcomes included in asset group analysis consistent (i.e. income measures), households receiving fewer or greater than 11 ducks were eliminated from duck asset group analysis. In sum, the number of households for each asset were 1233 for duck, 872 for chicken, and 2407 for vegetables.

Asset maintenance over 12 months was analyzed every three months. Differences in mean values with SD of each quarterly follow-up were determined using one-way ANOVA tests. The proportion of households who maintained or increased the initial asset provision was examined for each asset type. 2095 and 865 households were analyzed for duck maintenance and purchase. 872 and 2088 households were analyzed for chicken maintenance and purchase. 2403 households were examined for vegetable production.

Mean (±SD), median and interquartile range (IQR) for the annual income and consumption of chicken and duck groups were calculated. The linear regression model was used to calculate the β-coefficient with 95% confidence intervals (95% CI) for the association between the two asset groups, with the chicken group as a reference. On the basis of model 1, model 2 was additionally adjusted for the community location.

## Results

### Asset distribution

In total, 2125 households received ducks throughout 3- and 6-month follow-ups (Figure 2 and Table 1). 2095 households received ducks in Nov–Dec 2018 and 560 households received ducks in January–February 2019. 530 of the 560 households had previously received ducks at baseline and 30 of the households received their first and only batch of ducks after the 3-month follow-up. Among households receiving duck assets, the number of assets per household were 9 (n = 30), 11 (n = 1233), 17 (n = 375), 20 (n = 461), and 26 ducks (n = 69), with a mean duck asset provision of 14.4 ducks (Figure 2).

All households receiving chicken assets received 11 chickens (n = 872) at baseline. A total of 2407 households received vegetable seeds/gardening training. There was a significant overlap in the distribution of seeds with other assets. 607 (70%) households receiving chicken also received seeds, while 1822 (86%) duck households received seeds.

### Seasonal comparisons

#### Asset management

The numbers of assets present, born, died, consumed, and sold were found to be significantly different for duck, chicken, and vegetable groups across the follow-ups (p < 0.001, for all). The numbers of assets purchased were not significantly different across follow-ups (Table 2).

Total number (Mean (**±**SD)) of ducks present at the quarterly follow-up was 9.2 (5.7) at 3-month follow-up, then increased to 14.9 (10.0) at 6-month follow-up, and then decreased to 8.0 (8.1) at 9-month follow-up, and 6.2 (5.8) at 12-month follow-ups. Total number of chickens present at the quarterly follow-up was 9.0 (5.9) at 3 months follow-up, then increased to 16.9 (13.6) at 6-month follow-up. The number then decreased to 10.7 (10.8) at 9-month, and 8.2 (8.7) at 12-month follow-ups. Vegetable production declined from 26.8 (41.2) at 3 months, to 14.7 (39.8), 17.0 (39.3), and 20.1 (47.7) kgs at 6-, 9-, and 12-month follow-ups. Both duck and chicken groups reported peak asset per household at the 6-month follow-up, while the vegetable group reported highest mean asset at 3-month follow-up.

The highest number (**±**SD) of asset offspring born was found at 3-month follow-up for the duck group 2.5 (4.8) and tended to decrease over the course of 12 months. On the other hand, the chicken group tended to increase the offspring number and reported 3.2 (5.6) chicks at the 12-month follow-up. The highest number (**±**SD) of asset deaths was reported at 9-month follow-up for both groups – 4.5 (5.4) for the duck group and 4.3 (4.3) for the chicken group. No seasonal difference was observed regarding the asset purchase for both groups (p = 0.58 for duck group, p = 0.19 for chicken group).

The duck group increased their meat consumption over the quarterly follow-ups; a mean (**±**SD) of 0.2 (0.6), 0.6 (1.3), 1.9 (2.3), and 1.2 (2.4) ducks, at 3-, 6-, 9-, and 12-month follow-ups, respectively. However, the duck group’s egg consumption decreased gradually from 73.0 (99.8) to 59.1 (119.8), 47.2 (73.8), and 40.6 (59.9) duck eggs across the four respective follow-ups. Among the chicken group, both meat and egg consumption peaked at the 9-month follow-up: 2.8 (3.8) chickens and 35.9 (69.5) chicken eggs. Households with vegetable assets consumed a mean (**±**SD) of 15.0 (20.4), 7.8 (12.6), 10.7 (14.7), and 12.0 (19.8) kgs of vegetables across the four follow-ups.

Number of poultry assets (**±**SD) sold peaked at 9-month follow-up for both ducks (2.7 (4.7)) and chickens (3.2 (5.7)). Selling for the vegetable group (in kg) peaked at the 3-month follow-up (14.2 (40.0)).

#### Income

Significant seasonal trends in income were found among all asset groups – duck, chicken, and vegetable groups (p < 0.001, for all). Both ducks and vegetable groups reported peak income per household (**±**SD) at the 3-month follow-up: 1009 (1208) TK and 256 (730) TK, respectively, while the chicken group reported its highest mean income at the 9-month follow-up (753 (1244) TK).

#### Asset maintenance

Among households that received ducks as assets (n = 2095), the proportion of those maintaining duck presence was 97.0% at 3-month follow-up and decreased to 84.5% at 12-month follow-up. Notably, the proportion of households that did not receive duck assets but newly started duckling farming (n = 865) increased from 1.6% at 3-month follow-up to 18.3% at 12-month follow-up.

Among the households that received chickens as assets (n = 872), the proportion of participants maintaining chickens was 91.9% at 3-month follow-up and decreased to 79.8% at 12-month follow-up. The proportion of households that did not receive chicken as assets but started to possess chickens later increased from 7.0% at 3-month follow-up to 46.6% at 12-month follow-up. The proportion of households maintaining vegetable production increased throughout the quarterly follow-ups.

### Asset group comparisons

The annual income and consumption of the assets among the households with 11 ducks was compared to households receiving 11 chickens (Table 3).

#### Income

The difference in income between meat alone (log-transformed β of 1.15 (p = 0.003)), eggs alone (log-transformed β of 2.21 (p < 0.001)), and meat and eggs combined (log-transformed β of 1.65 (p < 0.001)) were all found to be significant between the duck and chicken asset groups throughout the study year. The combined mean annual earnings (**±**SD) from the selling of meat and eggs combined was higher for the duck group (3267 (3246) TK) than the chicken group (1930 (2321) TK). The mean annual income from meat sales alone was higher for the duck group (1480 (1892) TK) than the chicken group for (1283 (1655) TK). Concerning the poultry eggs, the mean income was higher from duck sales (1778 (2253) TK) than from chicken sales (657 (1376) TK).

#### Consumption

 Annual consumption of eggs was significantly higher in the duck group (223.5 (234.5) eggs) than in the chicken group (101.1 (138.6) eggs) (log-transformed β of 2.40 (p < 0.001)). Poultry consumption was lower in the duck group (3.8 (3.7) ducks) compared to the chicken group (6.6 (6.0) chickens) (log-transformed β of 0.084 (p < 0.001)).

## Discussion

Leveraging a year of monitoring data from a livelihood promotion program, this study evaluated the one-year impact of a graduation approach program in Bangladesh. The asset status of households with ducks, chickens, or vegetables showed a decrease in the number of assets per household between the second period and third follow-up period, while an increase in asset consumption during the monsoon season period. Moreover, the participating households reported voluntary income generation by purchasing additional assets, beyond the initial supply. In the analysis of asset value, households breeding ducks presented a better performance in income generation than those breeding chickens.

Our results aligned with findings from other similar studies in LMICs Ethiopia. A study reported beneficial outcomes of household agricultural practices, such as home gardening or livestock farming, including improvements in food security [19], dietary diversity [20,21], and nutritional status [22]. The vegetable production of the households reflected seasonal fluctuations in Bangladesh, where the lowest production and income were reported during the monsoon and pre-monsoon season.

The number of ducks (Mean: 4.5) and chickens (Mean: 4.3) dying reached its peak in July to September, Bangladesh’s rainy season. Multiple studies depicted poultry disease, including aflatoxicosis, nutritional deficiency, and infectious bursal disease, as major constraints for the poultry industry in Bangladesh [23,24]. Of note, previous investigation of the prevalence of poultry disease in the Narsingdi district of the country reported the highest incidence rate of poultry diseases during the monsoon season (47%), followed by summer (28%) season [25]. Given that the usual Bangladeshi diet fails to meet dietary recommendations for protein, specific training in poultry farming, an essential component of asset management, could offer opportunities for improving protein intake.

The national festival, *Eid al-Adha*, may also explain the gap between the second (Apr–June) and third (Jul–Sep) survey cycles in asset management. We observed the highest number of poultries sold for both duck (Mean: 2.7) and chicken (Mean: 3.2) households during the third survey cycle. Such trends may be due to the compensation mechanism to offset the temporal increase in expenditure during the Eid festival. During the most honored annual event for Muslim countries, Islamic people increase their expenses to purchase the festival goods [26] and slaughter a massive number of animals to celebrate and prepare holiday foods [27]. The rise of sales price due to the increased demand for meats during the festival season may have encouraged the participants to sell assets at a higher rate. We also observed the highest poultry consumption during the same period, where similar assumption indicates that the festival may necessitate greater intake of poultry assets.

The decline of food security during the monsoon season may be an alternate explanation for the highest meat and egg consumption among the poultry-breeding households from July to September. Prone to frequent flooding and food shortage, the peak monsoon season may alter the food availability of the households, through market price fluctuation and increased mortality of livestock [28]. The association between monsoon season and child nutritional status has been previously investigated globally [29,30]. A study examining 600 households in Dinajpur, Bangladesh, reported a higher prevalence of household food insecurity and child wasting during the monsoon season than in the dry season [28]. Decreased dietary diversity and increased unemployment may exacerbate child undernutrition during the rainy season. The ED program could offer an opportunity for households to earn year-round income, thus improving purchasing power for external food during seasons of vulnerability, while also providing access to direct food sources, so long as the asset base remains stable. However, additional program support should be available to households during this time to ensure that the program participants do not deplete their assets during seasons of vulnerability.

Regarding asset management, several aspects should be strengthened in future implementation: education to enhance breeding techniques, vaccination for poultry disease, support during the monsoon season, and education on asset management and asset depletion prevention. Further economic studies are suggested to support household asset management to meet optimal benefits from income generation and food security.

We observed a significant rise in poultry purchases among the counter asset group. Perhaps the increased demand for poultry and change in buying behavior among counter asset groups may indicate the ED program’s spill-over benefits, such as diversification of assets among participating households. The positive transmission effect of the technical, educational, or behavioral support of the development programs in the vulnerable population has been reported in multiple literatures [31,32]. Empirical evidence from India found a spill-over effect of education, where each additional year of education significantly increased household farm productivity. The World Food Programme (WFP) took a similar approach in targeting populations in northwest Bangladesh via its Food Security for the Ultra-poor (FSUP) project, reaching approximately 30,000 households [33]. The WFP study reported both intra- and inter-household spill-over benefits and improved nutritional status among the participating household children. Further, positive changes of the neighboring non-participants were found, including enhanced production capacity, asset management skills, knowledge transmission, increased health behavior and knowledge, women empowerment, and social networks [33]. The study outcome may support the previous suggestion by Kidd & Bailey-Athias [12], where the complex multi-step structure was highlighted in order to extend impact bonds throughout the community. While a year-long follow-up of the ED program showed positive behavior changes for income generation, further epidemiological studies with robust study designs are warranted to examine the long-term sustainability of program outcomes.

Duck assets appeared to be more effective at generating income than chicken assets. When comparing the poultry eggs, the mean income generated from ducks was more than double the income from chickens. The nutritional benefits of poultry have been investigated in various studies [34,35], where meat and eggs are dense in calories and protein, with ample amounts of vitamins and essential amino acids [36–38]. Of note, ducks may be an optimal option for the beneficiaries when considering the productivity and waterfowl habitat of Bangladeshi wetlands. A study examining the small-scale livestock farming in Kenya reported higher hatchability (82.3%) and fertility (89.5%) in ducks compared to chicken (66.2% and 82.8%, respectively) [39]. Further, another study of 100 farmers of rural Bangladesh observed maximum egg production during the rainy season, partially due to the greater availability of natural feed sources in ponds [40].

Poultry raising is a productive alternative for smallholder farmers, who cannot raise large livestock animals. The average Bangladeshi household raises approximately 7 chickens and 4 ducks [41,42]. Although duckling is an efficient dietary and income source, technical, feeding, disease, marketing, and environmental constraints hinder the optimum outcome of the practice [43]. Another cross-sectional survey of 771 coastal duck farms in Bangladesh observed 0.53 times lower odds of suboptimal egg production among educated farmers (p < 0.001) [44]. Our results align with accumulating evidence, where a holistic approach is needed to support the ultra-poor population. Our findings further suggest that appropriate interventions, such as rearing skills and livestock vaccinations, may improve the productivity and sustainability of the ED program. The mechanisms behind the benefits of household consumption of assets should also be considered.

Recently, the prolonged COVID-19 pandemic has exacerbated food insecurity in LMICs, where a significant association between income decline with lower household food security and dietary diversity was reported in Bangladesh [45]. An investigation among a Filipino population participating in the graduation program found enhanced household resilience regarding financial and food security during the pandemic [46]. Hence, the multi-dimensional interventions of the ED program may suggest a greater potential for sustained momentum during a crisis.

Our study has several strengths. First, the sample size included in the program was large, including 2960 households. Second, our analysis was based on repeated longitudinal measurements over one year. Furthermore, the study examined the asset investment behaviors among counter-asset groups, implying a positive spill-over effect throughout the community.

However, several limitations should be considered. First, the long-term effectiveness and sustainability of the ED program including asset management will require a multi-year analysis. While asset management is a key component to the sustainability of the ED program, our data showed a trend of asset depletion throughout the first year analysis. Second, the study only included ultra-poor households from three districts in the Rajshahi Division, which decreases the generalizability of the study outcomes to the rural population. As participant characteristics, knowledge in farming, and the agricultural environment may differ by region, additional studies considering the disparities should be considered. Third, the Eid festival and rainy season overlapped; hence, we could not separate the impact of rainy season and cultural events on the study outcomes. Moreover, all program activities, such as selling, consumption, and income, depended on program participants’ recall. The data might be biased toward favorable outcomes, as households may inflate actual consumption and selling numbers. Last, we are limited by the available data as the monitoring records did not assess demographic, baseline, and dietary information of the participants.

The evidence found in this analysis is important for organizations and donors who are interested in rural development. This study presents a year-long trend in the profit, consumption, death, and sales of the three asset types, using the GA protocols in rural Bangladesh. The findings from the current study suggest a multi-dimensional approach to enhance the livelihoods of the ultra-poor population in Bangladesh, and further recognizes the feasibility of the ED program to support the vulnerable population in the region. A quick turnaround of income is important for ultra-poor households to sustain their daily livelihoods. More evidence is warranted to examine the long-term effect of ED activities on household economies and food security. Further studies, including cohort, intervention, or randomized-controlled trials, are suggested considering the heterogeneity of demographic features, to make more robust comparisons between asset groups.

## Paper context

Multiple poverty eradication programs made significant economic achievements in Bangladesh. However, the ultra-poor population remains substantial, missed out from the conventional development scheme. A multifaceted economic development approach designed by World Vision was implemented to support the ultra-poor population to graduate the poverty status and move to an upward economic trajectory. The findings provide details for the feasibility and sustainability of the program to enhance the income generation and food availability among ultra-poor population.

## References

[1] World Bank. World bank open data. [cited 2021 Jun]. Available from: https://data.worldbank.org/country/bangladesh

[2] Khandker SR. Microfinance and poverty: evidence using panel data from Bangladesh. World Bank Econ Rev. 2005;19:263–12.

[3] Nazia Moqueet JZ, Isabel Whisson. Ultra-poor graduation handbook. 2019.

[4] Bangladesh Bureau of Statistics (BBS), Statistics and Informatics Division (SID). Ministry of planning. Bangladesh Statistics. Dhaka (Bangladesh); 2018.

[5] World Bank. Gini index (World Bank estimate). Bangladesh: The World Bank Group; 2016. Available from: https://data.worldbank.org/indicator/SI.POV.GINI?end=2019&locations=BD&start=1982. Jun 2021.

[6] Ferdousi S, Dehai W Economic growth, poverty and inequality trend in Bangladesh. 2014.

[7] National Institute of Population Research Training – NIPORT, Ministry of Health Family Welfare, ICF. Bangladesh demographic and health survey 2017-18. Dhaka Bangladesh: NIPORT/ICF; 2020.

[8] Waid JL, Sinharoy SS, Ali M, et al. Dietary patterns and determinants of changing diets in Bangladesh from 1985 to 2010. Curr Dev Nutr. 2019;3:nzy091. 30993255 10.1093/cdn/nzy091 PMC6459985

[9] Al Hasan SM, Saulam J, Kanda K, et al. Temporal trends in apparent energy and macronutrient intakes in the diet in Bangladesh: a joinpoint regression analysis of the FAO’s food balance sheet data from 1961 to 2017. Nutrients. 2020;12:2319. 10.3390/nu12082319 PMC7469017 32748820

[10] Magnani R, Oot L, Sethuraman K, et al. USAID office of food for peace food security country framework for Bangladesh (FY 2015–2019). Washington (DC): USAID; 2015.

[11] BRAC. BRAC’s ultra-poor graduation programme: an end to extreme poverty in our lifetime. Dhaka (Bangladesh): BRAC; 2017.

[12] Kidd S, Bailey-Athias D. The effectiveness of the graduation approach: what does the evidence tell us. Policy Focus. 2017;14:22–28.

[13] World Vision. Ultra poor graduation: world vision. [cited 2021 Aug]. Available from: https://www.wvi.org/economic-development/ultra-poor-graduation

[14] Sulaiman M Making sustainable reductions in extreme poverty: a comparative meta-analysis of livelihood, cash transfer and graduation approaches, 2016.

[15] Munshi Sulaiman NG, Dean K, de Montesquio A. Eliminating extreme poverty: comparing the cost-effectiveness of livelihood, cash transfer, and graduation approaches. Washington (DC): CGAP; 2016.

[16] Banerjee A, Duflo E, Goldberg N, et al. Development economics. A multifaceted program causes lasting progress for the very poor: evidence from six countries. Science. 2015;348:1260799. 25977558 10.1126/science.1260799

[17] Bandiera O, Burgess R, Das N, et al. Labor markets and poverty in village economies. Quarterly J Econ. 2017;132:811–870.

[18] Kang Y, Cho M, Rahman MM, et al. Design of a collaborative monitoring and evaluation system for a community-based nutrition project in rural Bangladesh. Eval Program Plann. 2021;84:101892. 33278719 10.1016/j.evalprogplan.2020.101892

[19] Yeudall F, Sebastian R, Cole DC, et al. Food and nutritional security of children of urban farmers in Kampala, Uganda. Food Nutr Bull. 2007;28:S237–46. 17658070 10.1177/15648265070282S203

[20] Jensen P. The urban gardens program for HIV-affected women and children: a review and look to the future. Washington (DC): FHI 360/FANTA; 2013.

[21] Masashua HE, Hawassi FGH, Dimoso PJ, editors. Potentials of urban horticulture for poverty reduction in Dar Es Salaam: a case of Kinondoni municipal; 2009.

[22] Mawoneke S, King B, editors. Impact of urban agriculture research in Zimbabwe 12000.

[23] Barua A, Yoshimura Y. Rural poultry keeping in Bangladesh. World Poul Sci J. 1997;53:387–394.

[24] Giasuddin M, Sil B, Alam J, et al. Prevalence of poultry diseases in Bangladesh. J Biol Sci. 2002;2:212–213.

[25] Uddin M, Ahmed S, Hassan M, et al. Prevalence of poultry diseases at Narsingdi, Bangladesh. Int J Biol Res. 2010;1:9–13.

[26] Khan MR, Sharma K. Purchase preferences and buying influences on religious occasions. FIIB Bus Rev. 2020;9:216–227.

[27] Noviyanti N. Implementing social marketing strategies to improve food-safety awareness during Eid-Al Adha festival in Indonesia. Proc Ind Focus. 2017;1:20.

[28] Hillbruner C, Egan R. Seasonality, household food security, and nutritional status in Dinajpur, Bangladesh. Food Nutr Bull. 2008;29:221–231. 18947035 10.1177/156482650802900308

[29] Panter-Brick C. Seasonal growth patterns in rural Nepali children. Ann Hum Biol. 1997;24:1–18. 9022902 10.1080/03014469700004732

[30] Trowbridge FL, Stetler HC. Results of nutritional status surveillance in El Salvador, 1975-77. Bull World Health Organ. 1982;60:433. 6982777 PMC2535995

[31] Gille V. Education spillovers: empirical evidence in rural India. Indian Growth Dev Rev. 2012;5(1):4–24.

[32] Hossain ME, Hoque MA, Giorgi E, et al. Impact of improved small-scale livestock farming on human nutrition. Sci Rep. 2021;11:1–11. 33420257 10.1038/s41598-020-80387-x PMC7794515

[33] Mamun-ur-rashid M, Khan SR Sustainability assessment of the impacts of the food security for the ultra poor (FSUP) project implemented by WFP in North-West Bangladesh.

[34] Ganesan P, Kaewmanee T, Benjakul S, et al. Comparative study on the nutritional value of pidan and salted duck egg. Korean J Food Sci Anim Resour. 2014;34:1. 26760738 10.5851/kosfa.2014.34.1.1 PMC4597835

[35] Sun C, Liu J, Yang N, et al. Egg quality and egg albumen property of domestic chicken, duck, goose, Turkey, quail, and pigeon. Poult Sci. 2019;98:4516–4521. 31287885 10.3382/ps/pez259

[36] Aronal A, Huda N, Ahmad R. Amino acid and fatty acid profiles of peking and muscovy duck meat. Int J Poul Sci. 2012;11:229–236.

[37] Marangoni F, Corsello G, Cricelli C, et al. Role of poultry meat in a balanced diet aimed at maintaining health and wellbeing: an Italian consensus document. Food Nutr Res. 2015;59:27606. 26065493 10.3402/fnr.v59.27606 PMC4462824

[38] Réhault-Godbert S, Guyot N, Nys Y. The golden egg: nutritional value, bioactivities, and emerging benefits for human health. Nutrients. 2019;11:684. 10.3390/nu11030684 PMC6470839 30909449

[39] Mbuthia P, Njagi L, Nyaga P, et al. Hatchability and fertility of Indigenous chicken and duck eggs, and some causes of chick and duckling mortality in Kenya. Kenvet. 2007;31:6–13.

[40] Pervin W, Chowdhury S, Hasnath M, et al. Duck production strategy and profile of duck farmers in the coastal areas of Bangladesh. Livestock Res Rural Dev. 2013;25:2013.

[41] Bangladesh Bureau of Statistics (BBS). Statistical Year book of Bangladesh. Ministry of planning, Government of the People’s Republic of Bangladesh, Dhaka: Bangladesh bureau of statistics. 2007, 33-4 p.

[42] Bangladesh Bureau of Statistics (BBS). Statistical Year book of Bangladesh. Ministry of planning, Government of the People’s Republic of Bangladesh, Dhaka: Bangladesh bureau of statistics. 2004.

[43] Rahman M, Khan M, Chowdhury S, et al. Duck rearing system in southern coastal districts of Bangladesh. Ban J Anim Sci. 1970;38:132–141.

[44] Hoque M, Skerratt L, Rahman M, et al. Factors limiting traditional household duck production in Bangladesh. Trop Anim Health Prod. 2010;42:1579–1587. 20517644 10.1007/s11250-010-9609-z

[45] Kundu S, Banna MHA, Sayeed A, et al. Determinants of household food security and dietary diversity during the COVID-19 pandemic in Bangladesh. Public Health Nutr. 2021;24:1079–1087. 33317657 10.1017/S1368980020005042 PMC8025083

[46] Karin Schelzig AJ. Assessing the impact of the graduation approach in the Philippines. Mandaluyong City: Asian Development Bank; 2021.

